# Strong and Ductile Electroplated Heterogeneous Bulk Nanostructured Nickel

**DOI:** 10.3390/ma12101573

**Published:** 2019-05-14

**Authors:** Yaoyao Jiang, Jun Yi, Kai Hu, Jing Zhao, Bo Huang, Yandong Jia, Gang Wang

**Affiliations:** Institute of Materials, School of Materials Science and Engineering, Shanghai University, Shanghai 200444, China; yaoyaojiang@shu.edu.cn (Y.J.); hk1217@shu.edu.cn (K.H.); xiaoxiaojing@shu.edu.cn (J.Z.); huangb@shu.edu.cn (B.H.); yandongjia@shu.edu.cn (Y.J.); g.wang@shu.edu.cn (G.W.)

**Keywords:** nanostructured nickel, electroplating, heterogeneous microstructure, ductility, work hardening

## Abstract

Porosity-free bulk nanostructured nickel cannot be fabricated by conventional electroplating due to hydrogen bubbling at the cathode. Here, we developed a cathode-rotating electroplating technique to remove the bubbles in order to obtain millimeter-scale nanostructured nickel rods with low porosity. The grain sizes ranged from 20 to 300 nm. The range produced by the new technique was broader than those that have been reported. The heterogeneous microstructure contributed to high work hardening rate, yield strength, and ductility of the rods in tension. The ductility was larger than electroplated thin nickel film with comparable ultimate strength in the literature. Dislocations accumulated at pre-existing twins, grain boundaries, and at the grain interior mediated the plastic deformation of the rods.

## 1. Introduction

Nanostructured metals with nanocrystalline and ultrafine grains exhibit increased hardness [1,2,3,4,5,6], ultrahigh yield and fracture strength [1,3,7,8,9], superior wear resistance [3,5,10], and superplasticity at cryogenic temperatures [1,11] compared with their coarse-grained counterparts. These attributes have stimulated extensive research interest in their structural engineering applications. Bulk nanostructured metals can be synthesized through several approaches involving the synthesis and consolidation of nanoparticles, such as inert gas condensation [1], or of nanocrystalline powders, such as ball milling [12] and severe plastic deformation [13]. Bulk porosity-free nanostructured metals can be synthesized by severe plastic deformation, whereas the typical grain sizes obtained by this approach still remain in the range of 150–300 nm or larger [14]. The nanostructured metals prepared by this technique exhibited near-zero uniform tensile ductility owing to their low rate of work hardening [9]. Although a number of strategies have recently been developed to improve the poor mechanical properties in nanostructured metals, such as the introduction of gradient nano-grains [15,16,17,18,19], pre-existing growth nano-twins [20], and the formation of second-phase particles [21], most of them retain some inherent and material limitations [14,22].

Electroplating is an old technique that has developed into an important branch of nanotechnology. This technique has been used to produce nanostructured metals [23,24]. For electroplated nanostructured metals, the microstructure and properties can be tuned by electroplating parameters [25]. Porosity-free bulk nanostructured metals with a superior combination of high strength and high ductility have been fabricated by this technique [26]. The fabrication of bulk nanostructured nickel prepared by this technique was reported [27,28,29], while the porosity of the materials was not investigated. However, porosity-free bulk nanostructured nickel cannot be processed by electroplating, because the discharged hydrogen atoms form bubbles at the cathode surface during electroplating [30], and nickel atoms cannot be electroplated at the bubbles. Specific surfactants can reduce the adhesion between hydrogen bubbles and cathode substrate, but they cannot completely eliminate the bubbles [30]. Due to the bubble defects, no bulk electroplated nickel with a combination of high strength and high ductility has been reported to the best of our knowledge. The maximum thickness of porosity-free nanostructured nickel prepared by electroplating is about 500 μm, and significantly limits its applications. We have noticed that rotating cathode substrates are commonly used in electroplating industry and research, while to our best knowledge people have not attempted to eliminate hydrogen bubbles by using such substrates.

Here, we introduce a cathode-rotating electroplating technique to remove hydrogen bubbles by centrifugal force in order to obtain bulk nanostructured nickel with low porosity. The traditional Watts electrolyte and a constant current power supply were chosen to demonstrate the effect of the bubble removal.

## 2. Materials and Methods 

**Figure 1:**
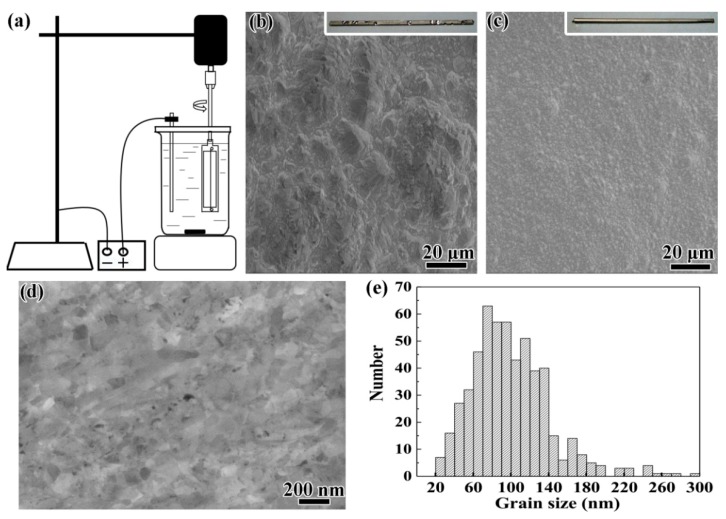
(**a**) Schematic illustration of the cathode-rotating electroplating technique. (**b**,**c**) SEM images of the surface morphology of static and rotated cathodes with electroplated nickel, respectively; the insets are macro images of the macroscopic morphology. (**d**) The cross-sectional microstructure of the electroplated bulk nanostructured nickel with a diameter of 1 mm. (**e**) The statistical distribution of grain size which was obtained from five SEM images like the one shown in Figure 1d.

Our cathode-rotating electroplating setup is shown in Figure 1a. This is a revised version of a classical electroplating setup. A servo-motor was introduced to rotate the nickel wire cathode which was fixed and stretched on an epoxy resin frame, which in turn was fixed onto the motor through a stainless-steel rod (Figure 1a). The diameter of the nickel wire was 20 μm. During electroplating, the rotating speed of the servo-motor was set as 1300 rpm. The rotating can not only achieve homogeneous electroplating onto the wire as demonstrated in our previous work [31], but can also remove hydrogen bubbles. A pure nickel (purity: 99.99%) plate with dimensions of 2 mm × 10 mm × 100 mm was used as an anode. The lengths of the parts of the wire and the pure nickel anode immersed in the electrolyte were 60 mm. 

The Watts electrolyte that we used was composed of 0.43 M NiSO_4_·6H_2_O, 0.1 M NiCl_2_·6H_2_O, 0.24 M H_3_BO_3_, and deionized water. A magnetic stirrer was used to stir the electrolyte during electroplating. A constant-current power supply (Keithley 2230G-30-1, Tektronix, Cleveland, OH, USA) was used to provide a constant current with an initial current density of 2 mA·mm^−2^. Nanostructured nickel rods with diameters of 1 mm were fabricated at room temperature in 57 h. Grip sections of tensile testing samples were prepared by immersing end sections of the rods in the electrolyte, applying reversed current to remove oxidized surface layer for 10 s, and then electroplating nickel with a constant current and an initial current density of 2 mA·mm^−2^. The preparation of one grip section took 48 h. The ratio of the gauge length to gauge diameter was kept at a constant value of 5 throughout all tensile testing samples. After the preparation, the samples developed a smooth fillet between the gauge sections and the grip sections, as reported in our previous work about copper electroplating [31].

Tensile tests of the nanostructured nickel samples were carried out on an MTS Landmark servo hydraulic test system (MTS, Eden Prairie, MN, USA) with a 370.10 load frame and a FlexTest 40 controller. An extensometer (Central Iron and Steel Research Institute YYU-5/10, Beijing, China) was used to measure strain. The tensile tests were performed in displacement control mode at room temperature.

In order to investigate the microstructure of the samples, cross sections were prepared by diamond wire saw (Well diamond wire saw 3500+ Premium version, WELL Diamond Wire Saws, Inc., Norcross, GA, USA) cutting, SiC paper grinding, and vibratory polishing (Buehler VibroMet 2 vibratory polisher, Buehler, Lake Bluff, IL, USA) with a 40-6380-064 non-crystallizing colloidal silica polishing suspension. Microstructure and deformation morphology of the electroplated nanostructured nickel were investigated by a Carl Zeiss GEMINI 300 scanning electron microscope (SEM, Carl Zeiss AG, Oberkochen, Germany) and a JEM-2010F transmission electron microscope (TEM, JEOL, Tokyo, Japan). An FEI (600i, Hillsboro, OR, USA) focused ion beam (FIB) system was employed to prepare TEM samples with dimensions of 3.5 μm × 3.5 μm × 100 nm from the polished cross section.

## 3. Results

### 3.1. Microstructure

To explore the effect of cathode rotation, the morphology of static and rotated cathodes with deposited nickel with diameters of 1 mm are shown in Figure 1b,c, respectively. No macroscopic and microscopic pore was observed on the surface of electroplated bulk nickel on the rotated cathode, while electroplated nickel on the static cathode was porous both microscopically and macroscopically. In addition, the surface of the electroplated bulk nickel on the rotated cathode was much smoother.

In order to further investigate the porosity of the electroplated nickel fabricated by the cathode-rotating electroplating technique, cross-sectional morphology was investigated as shown in the SEM image in Figure 1d. Figure 1d shows few pores with diameters of about 20 nm at grain boundaries. Figure 1d also shows the microstructure of the bulk electroplated nanostructured nickel. Figure 1e presents the grain size distribution, which was determined by a linear intercept method, in the cross-sectional SEM images of five nickel rods with diameters of 1 mm fabricated by the cathode-rotating technique. The grain size ranged from 20 to 300 nm, while the average grain size was calculated to be 86 nm. The grain size distribution was much broader than the reported values for electroplated nickel [14,32,33]. Therefore, the yielding of the materials cannot be predicted by a Hall–Petch relationship because of the heterogeneous microstructure. The special microstructure may be produced by varying the Helmholtz compact double-layer thickness induced by varying the contact electrical resistance between the brush and the rotor in the motor.

### 3.2. Mechanical Behaviours

**Figure 2:**
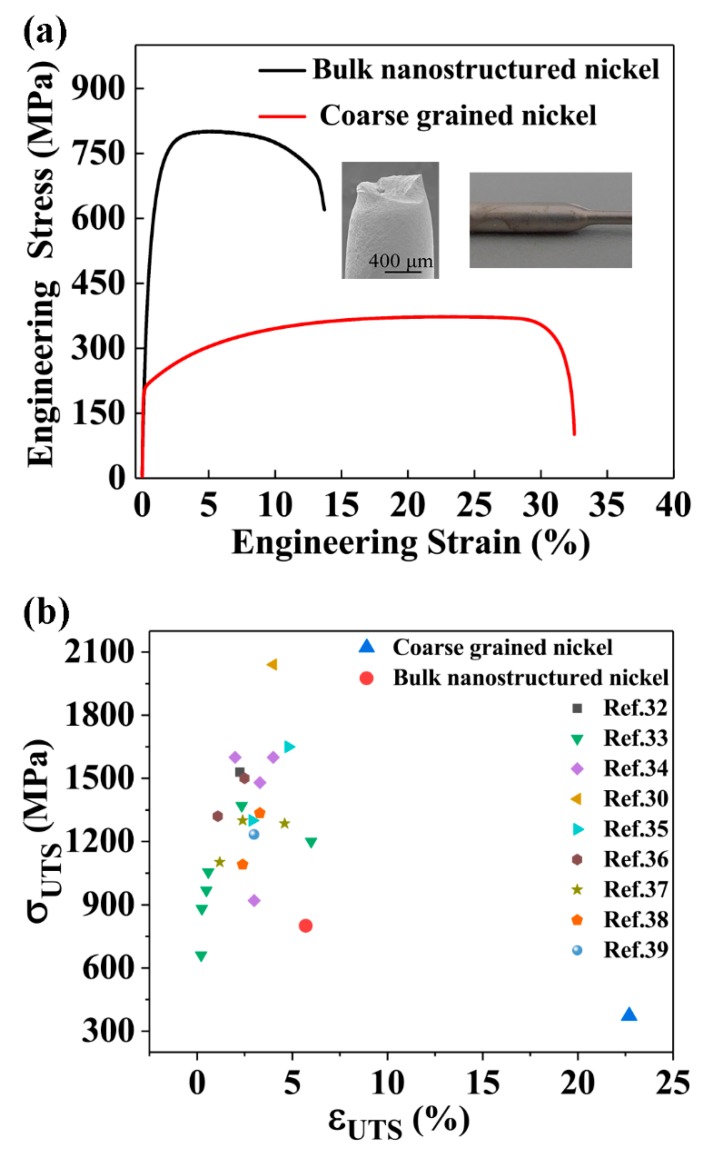
(**a**) Engineering tensile stress–strain curves of bulk electroplated nanostructured nickel and coarse-grained nickel (strain rate = 1 × 10^−3^ s^−1^). (**b**) Compilation of ultimate strength versus elongation at the ultimate strength of the bulk electroplated nanostructured nickel with a diameter of 1 mm, the coarse-grained sample, and electrodeposited nickel thin film in [32,34,35,36,37,38,39,40,41].

In order to further investigate the deposition quality of the bulk electroplated nanostructured nickel rods with a diameter of 1 mm, tensile tests were carried out at a strain rate of 1 × 10^−3^ s^−1^ on the materials and the engineering stress–strain curve is shown in Figure 2a. The engineering stress–strain curve of the coarse-grained nickel that was used as our anode material is also shown in Figure 2a for comparison. The inset SEM image shows necking of the tensile-tested sample, illustrating the deformation and fracture behavior of the sample. On the other hand, the inset optical image shows a smooth fillet between the grip section and gauge section, which is expected for a standard tensile test.

The 0.2% offset yield strength $\sigma_{y}$, the ultimate tensile stress $\sigma_{UTS}$, and the plastic strain at the ultimate tensile stress $\varepsilon_{UTS}$ were 541 MPa, 800 MPa, and 5.8%, respectively. Those of the coarse-grained nickel were 210 MPa, 373 MPa, and 22.9%, respectively. The nanostructured nickel with sacrificed ductility was much stronger than the coarse-grained nickel. When the combination of $\sigma_{UTS}$ and $\varepsilon_{UTS}$ was put into Figure 2b and compared with the values for electroplated nickel thin films with static cathodes in [32,34,35,36,37,38,39,40,41], we can see that the ductility was much larger than that for the thin films with comparable $\sigma_{UTS}$. This demonstrates that the amount of porosity of the bulk nanostructured nickel cannot be much higher than that of thin nickel films with a thickness range from 100 to 400 μm and grain sizes ranging from 19 to 350 nm in [32,34,35,36,37,38,39,40,41], which are comparable to the grain sizes of our samples. In addition, the grain size distributions in these works were much narrower than that in the present work.

### 3.3. Work Hardening Rate

**Figure 3:**
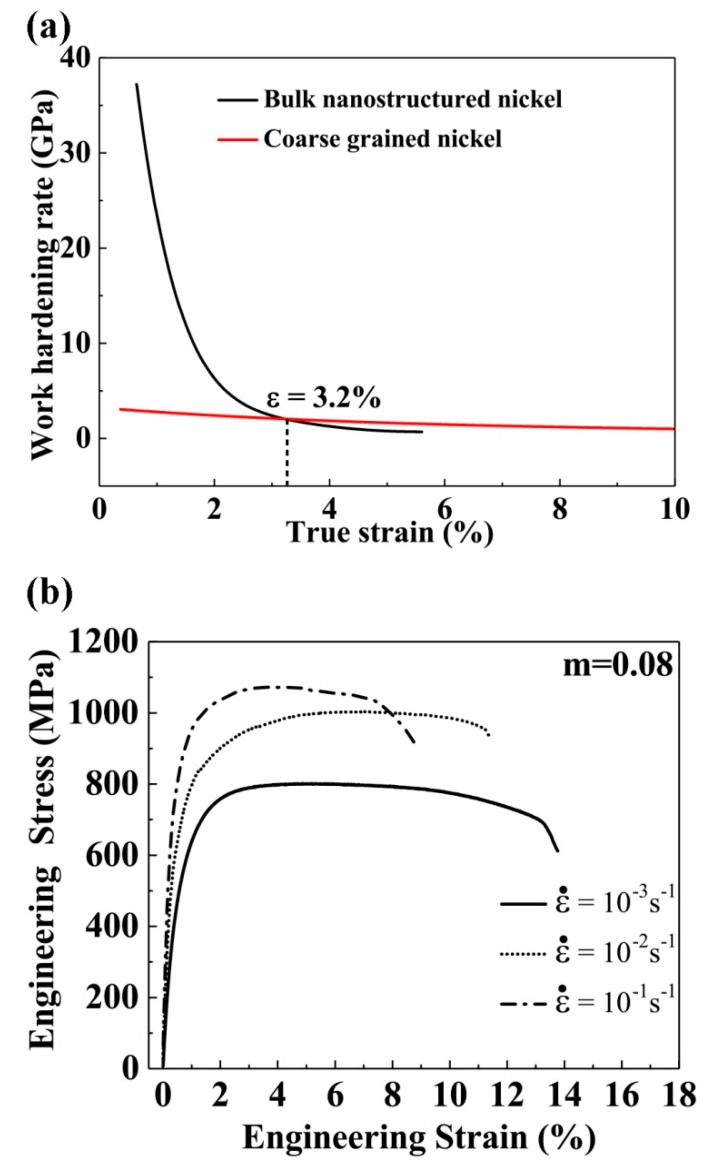
(**a**) Work hardening rate as a function of true strain for the bulk electroplated nanostructured nickel with a diameter of 1 mm and the coarse-grained nickel sample. (**b**) Tensile engineering stress-strain curves of the bulk electroplated nanostructured nickel at different strain rates. The strain rate sensitivity *m* of the nanostructured nickel was calculated to be 0.093.

The work hardening rate θ=dσ/dε (*σ* and *ε* are true stress and true strain, respectively) of the bulk electroplated nanostructured nickel was calculated and plotted as a function of true strain and compared with that of coarse-grained nickel, as shown in Figure 3a. It was obvious that the bulk electroplated nanostructured nickel exhibited much higher θ after yielding than that of coarse-grained nickel. After 3.2% plastic deformation, their θ became comparable. Usually, θ in nanomaterials is low due to the small grain size [14,22]. The higher θ of the electroplated bulk nanostructured nickel may have been due to its broad grain size distribution. 

Figure 3b shows engineering stress–strain curves obtained from the bulk electroplated nanostructured nickel deformed at four different strain rates. The plastic flow stress increased with increasing strain rate, while ductility decreased. From Figure 3b, the strain rate sensitivity, $m=\partial ln\sigma /\partial ln\dot{\varepsilon }$ ($\dot{\varepsilon }$ is strain rate), of the tested samples was calculated to be 0.093, which is remarkably higher than the values of the previously reported nanocrystalline and coarse-grained Ni: 0.01–0.03 and 0.001–0.004 [32,33], respectively. Usually, the strain rate sensitivity of metals increases with decreasing grain size. Here, the strain rate sensitivity was even much higher than that of nanocrystalline nickel with a grain size of 20 nm [32]. This may have been due to the heterogeneous microstructure. 

The combination of high *θ* and high *m* contributed to the high uniform elongation shown in Figure 2, because metal necking is determined by *θ* + *m* [42]. The bulk nanostructured nickel rods necked at the engineering strain of 5.8%, *θ* + *m* = 0.77. According to the Hart criterion, necking occurs when *θ* + *m* < 1. Therefore, our experimental results were consistent with Hart’s theory [42].

### 3.4. Deformation Mechanism

**Figure 4:**
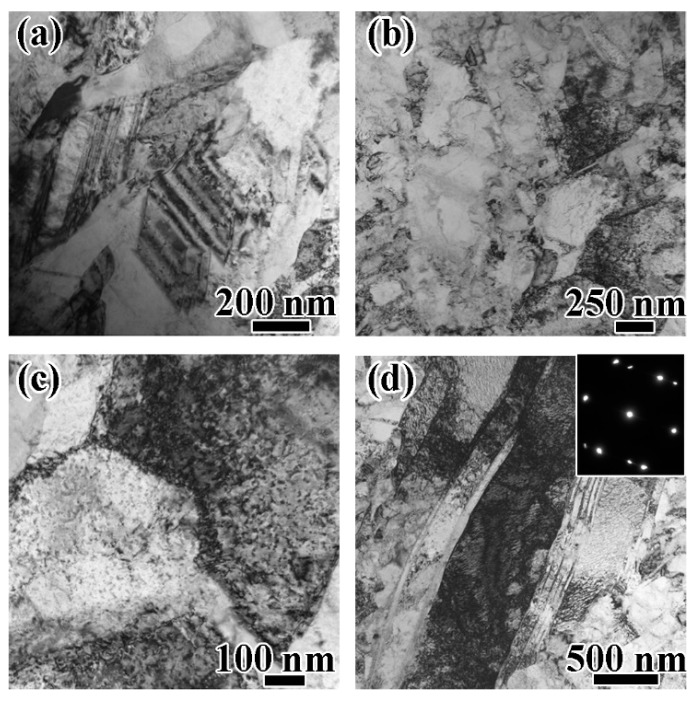
TEM investigation of plastic flow units of the bulk electroplated nanostructured nickel. (**a**) TEM image of as-plated nanostructured nickel. (**b**) Microstructure of post-deformed nanostructured nickel. (**c**) Dislocations accumulated at grain interior and grain boundaries in post-deformed nanostructured nickel. (**d**) Dislocations accumulated at growth twins in post-deformed samples. The inset shows the selected area diffraction pattern, and confirms the nature of the twins.

The microstructures of the bulk electroplated nanostructured nickel before and after tensile tests were examined with TEM to reveal the deformation mechanisms of the bulk electroplated nanostructured nickel. The TEM images of FIB samples which were taken from the cross-section of the nanostructured nickel rod are shown in Figure 4. 

Figure 4a shows the heterogeneous microstructure of the as-plated bulk nanostructured nickel. Straight growth twins and low-density pre-existing dislocation can be seen in the figure. Figure 4b–d shows some details after tensile tests. Figure 4b,c shows that a large number of dislocations accumulated at the grain boundaries. At the same time, grain interior dislocations existed in grains with size larger than 100 nm, and could not be found in smaller grains. This is consistent with results that have been reported elsewhere [22,43]. Both dislocations accumulated at grain boundaries and at the grain interior can contribute to the high work hardening rate [43] in Figure 3a. 

Figure 4d shows high-density dislocations accumulated at deformed growth twins. The accumulation demonstrated work hardening and ductility contributes of growth twins [20]. However, we did not find stacking faults, which have a positive effect on both strength and ductility. Therefore, plastic deformation of the bulk nanostructured nickel was dominated by dislocations as coarse-grained nickel [32,33].

## 4. Discussion

The combination of high yield strength and high ductility in the bulk electroplated nanostructured nickel may originate from low porosity and the heterogeneous microstructure shown in Figure 1. Low porosity mitigates stress concentration and hence increases fracture strength and ductility [44]. At the same time, heterogeneous microstructure may cause mechanical incompatibility, because dislocation needs higher stress to slide in smaller grains [14]. Therefore, smaller grains may deform elastically while larger grains deform plastically. This kind of mechanical incompatibility can change the stress state in the tensile testing samples and cause stress and strain gradients [45]. Geometrically necessary dislocations can accumulate to accommodate the gradients, and hence increase strength and work hardening rate [45,46], as shown in Figure 3a.

## 5. Conclusions

Bulk nanostructured nickel with superior mechanical properties was successfully fabricated with a cathode-rotating electroplating technique. This new technique could be extended to other metal (such as Sn, nickel alloys, and so on) electroplating during which bubbles are generated at cathode. Bulk samples with larger size can also be fabricated by the technique if more electroplating time is used. Therefore, we believe that our work can further extend application of electroplated metals. The technique could also be further developed to fabricate new bulk materials, such as nano-gradient metals and so on. Our new technique can produce a broader grain size distribution than other techniques that have been reported. Furthermore, we found that the mechanical incompatibility caused by broad grain size distribution could enhance the strength and ductility in nanostructured nickel. In a word, our work shed some light on tailoring the strength and ductility of bulk electroplated metals in order to obtain a better combination.
